# Supplementary material for: Complexome profiling on the Chlamydomonas lpa2 mutant reveals insights into PSII biogenesis and new PSII associated proteins
Source: J Exp Bot. 2021 Aug 26;73(1):245–62. doi: 10.1093/jxb/erab390 (PMC8730698; doi:10.1093/jxb/erab390)
Supplement: erab390_suppl_Supplementary_Dataset_S1 [file erab390_suppl_supplementary_dataset_s1.zip › Supplemental Dataset 1 - Excel List and all profiles/plots/NDA3_Cre05.g232200.html]

Trivial name: NDA3  
  
Euclidean distance: 46699.83  
Mean Intensity (WT): 6230.85  
Mean Intensity (Mut): 3550.39  
Distance: 7.49  
  
MapMan: mitochondrial electron transport / ATP synthesis.NADH-DH (type II).external;mitochondrial electron transport / ATP synthesis.NADH-DH.type II.internal matrix;mitochondrial electron transport / ATP synthesis.NADH-DH.type II.external  
  
p value of intensity sums Welch test: 0.0411
